# Intra-cranial Cephalhæmatoma

**Published:** 1886-01

**Authors:** E. S. McKee

**Affiliations:** Cincinnati


### Intra-cranial Cephalhæmatoma.

By E. S. McKee, M. D., Cincinnati.

The following is an abstract of a paper read before the Mississippi Valley Medical Society, September, 1885:

"A physician's library will, in many instances, give him no information regarding cephalhæmatoma. A large list of text-books may be searched, to no avail, for a mention of intra-cranial cephalhæmatoma. If the searcher after medical knowledge has a well-ordered and extensive public library at hand, well supplied with bound journals, search may be rewarded by a few hidden points."

The writer described cephalhæmatoma as an "effusion of blood occurring in newly-born infants, forming a tumor of the head." The intra-cranial variety he divided into those situated between the skull and dura mater and those situated in the arachnoid cavity.

Etiology he found to be very similar to that of the extra cranial variety. Pressure, during delivery, he considered to be a frequent cause, though the tumors occurred in children ot easy labors, the breech presentation, and those delivered by Cæsarian section. He thought the crushing during delivery would loosen the pericranium or dura mater from the bone and rupture the small bone blood vessels. This pressure being removed, the vessels having been relieved by diapedesis caused by the pressure, we have a vacuum. From her well-known *horror vacuii*, nature immediately proceeds to fill up this space.

She goes at it with such energy that she overdoes the matter, and hyperæmia and engorgement ensue. The sources of this hemorrhage are probably the tender blood vessels, their varicose condition, the hemorrhagic diathesis. Inherited tendency, or fissure of the bone, may be among the causes. The excess of the external variety over the internal is due, he thought, to the direction of the pressure, and to the greater porousness of the outer part of the skull.

*Diagnosis.*—This depends entirely on the symptoms of brain pressure, twitchings, convulsions, stupor and paralysis.

*Prognosis.*—Grave; death comes from brain pressure, necrosis, or carries off the bone leading to perforation; thrombosis of the cerebral sinuses; extension of the inflammation on to the meninges of the brain itself, and pyæmia; idiocy may result.

Dr. West found the repair of the internal variety very analogous to that of the external. After diligent search through all the journals, twenty cases were found mentioned.

Drs. West, Hennig, Von Siebold, Jackson, Held, Betscher, Cleveland, Ruge, Dubois, Padieu, Hoere, Bouchard, and the writer himself, have reported cases.

Treatment did not occupy much space in the paper, as the doctor said it had never yet been attempted in this variety, the diagnosis having always been made post mortem. He suggested the possibility of the trephine being of utility.

